# BRD7 inhibits tumor progression by positively regulating the p53 pathway in hepatocellular carcinoma

**DOI:** 10.7150/jca.50293

**Published:** 2021-01-01

**Authors:** Chang-Long Chen, Hua-Qian Mo, Yan-Hui Jiang, Xiao-Hui Zhao, Shuang Ma, Kai-Yun You, Yue Pan, Yi-Min Liu

**Affiliations:** 1 Guangdong Provincial Key Laboratory of Malignant Tumor Epigenetics and Gene Regulation, Medical Research Center, Sun Yat-Sen Memorial Hospital, Sun Yat-Sen University, Guangzhou, P. R. China; 510120; 2 Department of Radiation Oncology, Sun Yat-sen Memorial Hospital, Sun Yat-sen University, Guangzhou, P. R. China; 510120; 3 Center for Precision Medicine, Sun Yat-Sen University, Guangzhou, 510080, P. R. China; 510120

**Keywords:** BRD7, hepatocellular carcinoma, tumor suppressor, p53 pathway, transcriptional regulation

## Abstract

**Background:** Bromodomain-containing protein 7 (BRD7) is identified as a transcriptional regulator and plays an important role in the development and progression of various tumors. Our previous study demonstrated that BRD7 acts as a potential tumor suppressor in hepatocellular carcinoma (HCC). However, the specific molecular mechanism underlying the BRD7-mediated inhibition of HCC progression remains poorly understood.

**Methods:** We performed ChIP-seq analysis to investigate the gene network mediated by BRD7. Immunohistochemical analysis was performed to analyze potential associations between the p53 and BRD7 expression and the effect of their overexpression on disease pathogenesis and outcome. In addition, we performed biological function experiments to determine the effect of BRD7 and p53 on these functions that are central to tumorigenesis. Finally, we employed a BALB/c model for execution of xenograft transplants to examine the effect of either overexpressing or under-expressing BRD7 and p53 on tumor growth in mice injected with cells.

**Results:** Our results suggested that BRD7 regulates the p53 pathway. Specifically, BRD7 was demonstrated to upregulate the transcription level of p53 by directly binding to the upstream regulatory region of the p53 transcriptional initiation site, thereby enhancing its promoter activity. Moreover, immunohistochemical analysis showed that wild-type p53 (WTp53) expression is positively associated with BRD7 expression and survival of patients with HCC. Additionally,changes of p53 expression could affect the tumor suppressive role of BRD7 on HCC cell proliferation, migration/invasion, cell-cycle, and tumor growth *in vitro* and *in vivo*. Furthermore, changes of BRD7 expression in HCC cells significantly altered the expression of p53 signal-related molecules such as p21, Bax, Bcl2, and cyclin D1, indicating that BRD7 may positively regulate activation of the p53 pathway.

**Conclusions:** Collectively, our results indicated that BRD7 exerts anti-tumor effects in HCC through transcriptionally activating p53 pathway. These critical roles of BRD7may provide some promising diagnostic and therapeutic targets for HCC.

## Introduction

Hepatocellular carcinoma (HCC) is one of the most common malignant tumors worldwide, ranking 7^th^ in incidence, and 4^th^ in mortality among all solid tumors 1. Despite recent advances in diagnostic and therapeutic options, which have markedly improved the prognosis for early HCC, most patients continue to be diagnosed with advanced stage disease and ultimately require systemic therapies 2. Recently, molecular targeted drugs (e.g., sorafenib, cabozantinib, and ramucirumab), and immune-checkpoint inhibitors were incorporated into HCC treatment strategies and have demonstrated improved clinical outcomes 3,4. However, the overall prognosis for many patients with advanced HCC remains poor even with these aggressive interventions 5,6. The molecular mechanisms underlying the initiation of HCC development and malignant progression remain unclear 5.Therefore, it is essential to explore sensitive biomarkers and novel therapeutic strategies capable of improving the outcomes for this devastating disease.

Bromodomain-containing protein 7 (BRD7), first cloned from nasopharyngeal carcinoma cells, is involved in multiple physiological processes via mediation of chromatin remodeling 7-9. Accumulating evidence has demonstrated that when *BRD7* is downregulated it becomes associated with negative clinical outcomes in multiple types of malignant tumors, including breast cancer, colorectal cancer, and non-small cell lung cancer, suggesting that *BRD7* may serve as a tumor suppressor gene, thereby interfering with efficient cancer development and progression 10,11. Our previous study demonstrated that BRD7 expression is significantly associated with a favorable prognosis in HCC patients and is involved in impeding tumor development by inhibiting proliferation, cell cycle progression, and cellular migration/invasion 12. More recent studies have suggested that BRD7 inhibits tumorigenicity through multiple mechanisms. For example, BRD7 has been shown to arrest cell cycle progression in G1 through suppression of the Ras/MEK/ERK, ERK1/2, and β-catenin pathways 13,14. BRD7 may also interfere with breast cancer progression through inactivation of the HIF1α/LDHA transcriptional axis and via promotion of BRCA1-mediated transcriptional regulation of the estrogen receptor expression 10,15. Furthermore, BRD7 has been shown to inhibit cancer cell growth and metastasis by not only impairing the activity of the c-Myc/miR-141 transcriptional axis but also by downregulating PI3K/Akt-mediated survival signaling 16,17. Moreover, studies have demonstrated that BRD7 functions as a transcriptional coactivator for Smads, which promotes TGF-β-induced inhibition of cell growth 18. BRD7 was also shown to have an anti-inflammatory role during early acute inflammation via interference with activation of the NF-кB signaling pathway, which indicated that BRD7 may affect tumor development through regulation of the inflammatory components in the tumor microenvironment 19. However, the precise molecular mechanism underlying the tumor suppressive effect of BRD7 in HCC progression, specifically, has not yet been clearly elucidated.

In this study, therefore, we first performed chromatin immunoprecipitation sequencing (ChIP-seq) in BRD7-overexpressing HCC cells to identify the specific signaling pathways directly associated with BRD7. We determined that BRD7 is involved in the p53 pathway, and that it directly promotes transcriptional regulation of p53. To decipher this association further we detected p53 and BRD7 protein levels in HCC tissues and HCC cell lines. Furthermore, we investigated whether the p53 pathway is involved in BRD7-mediated suppression of HCC cell proliferation and tumor growth both *in vitro* and *in vivo* within a BALB/c mouse model. We found that the tumor suppressive effect of BRD7 is dependent on the p53 pathway through regulation of molecules directly involved in p53 signaling, namely, p53, p21, Bcl2, Bax and cyclin D1. To our knowledge, this study is the first to indicate that BRD7 inhibits HCC progression through activation of the p53 transcriptional axis.

## Materials and methods

### Cell cultures and cell transfections

HCC cell lines (Bel7402 and HepG2) were purchased from American Type Culture Collection (Manassas, VA, USA) in 2018/10 and thereafter not authenticated, but were routinely tested for mycoplasma contamination every 3 months. All the cell lines were cultured in Roswell Park Memorial Institute (RPMI) 1640 media supplemented with 1% penicillin-streptomycin, and 10% fetal bovine serum (FBS) at 37 ºC in a humidified incubator containing 5% CO_2_. The small interfering RNA (siRNA) and short hairpin RNA (shRNA) plasmid vectors for knockout of *BRD7* and *p53* were synthesized by GenePharma (Suzhou, China). The siRNA sequences are presented in table S1. HepG2 cells were transfected with siBRD7 or BRD7 shRNA (shBRD7), and Bel7402 cells were transfected with sip53or p53 shRNA (shp53) using Lipofectamine RNAiMax reagent (Invitrogen , Carlsbad, CA, USA) according to the manufacturer's protocol. Overexpression of p53 was accomplished using a plasmid (EX-B0105-M02-5) and a control plasmid (EX-eGFP-M02) was also obtained from Genecopoeia (Rockville, MD, USA). For restoration of p53, HepG2 cells were transfected with the p53 plasmid using Lipofectamine 2000 according to the manufacturer's instructions. Following infection for 48 h, the cells transfected with shRNA or p53 plasmid were selected by Geneticin (G418, Sigma, Louis, MO). Stable G418-resistant cells were pooled and cultured for tumorigenicity assays. Recombinant lentiviruses overexpressing BRD7 or empty control vectors were used to generate stable BRD7-overexpressing Bel7402 cells as described in our previous study 12. Cell lines were altered as follow: Bel7402 cells co-transfected with vector and siContorl, BRD7 and siContorl, BRD7 and sip53 were designated as Bel7402/Contorl, Bel7402/BRD7, and Bel7402/BRD7+sip53, respectively. Bel7402 cells co-transfected with vector and control shRNA(shContorl), BRD7 and shContorl, BRD7 and shp53 were designated as Bel7402/Contorl-1, Bel7402/BRD7-1, and Bel7402/BRD7+shp53, respectively. HepG2 cells co-transfected with siContorl and control plasmid, siBRD7 and control plasmid, siBRD7 and p53 were designated as HepG2/Contorl, HepG2/siBRD7, HepG2/siBRD7+p53, respectively. HepG2 cells co-transfected with shContorl and control plasmid, shBRD7 and control plasmid, shBRD7 and p53 were designated as HepG2/Contorl-1, HepG2/shBRD7, HepG2/shBRD7+p53, respectively. Transfection efficiency was validated by western blot analysis.

### ChIP-seq analysis and ChIP-qRT-PCR validation

All standard protocols for ChIP-seq, including sequence library preparation, sequencing, and quality control were performed by Novogene (Beijing, China). Briefly, cross-linked chromatin was sonicated, and purified from the HCC cell lines with and without immunoprecipitation for ChIP and the corresponding input DNA fragments, respectively. DNA fragments were sequenced using Illumina HiSeq2000 system (Illumina, San Diego, CA, USA) and were subsequently mapped onto the reference genome. The genomic regions, which were significantly enriched with ChIP reads compared to the input reads, were identified as peaks. Other genomic regions were regarded as non-specific background. Called peaks, which represent candidates for targeted protein/DNA-binding and histone modification sites, were used to identify associated functional annotations, including binding motifs and gene ontology. The ChIP-seq findings were validated by ChIP-PCR. In brief, ChIP was performed according to the Magna ChIP protocol (Millipore, Burlington, MA, USA) in Bel7402 cells transfected with BRD7, HepG2 cells transfected with siBRD7 or control cells using FLAG-tagged BRD7 antibody (Cell Signaling Technology, Danvers, MA, USA) and equivalent amounts of mouse IgG as a negative control. The purified IP DNA was then used for regular qRT-PCR. The validated genes and corresponding PCR primers were as follows: WTp53, 5′-GACAGCAGTCCGGAGC TAAC-3′ (forward) and 5′-TGCCTACTCCCAGAAGAGGA-3′ (reverse). GAPDH, 5′-CTCCTCCTGTTCGACAGTCAGC-3′ (forward) and 5′-CCCAATACGACCAAA TCCGTT-3′ (reverse). The qRT-PCR results were assessed via agarosegel electrophoresis.

### Dual-luciferase reporter assay

The WTp53 promoter region was amplified and cloned into the pGL3-luc vector to construct a p53-luc vector. Additionally, Bel7402 cells were transfected with BRD7-overexpressing lentiviruses or with empty control vectors and HepG2 cells were transfected with shBRD7 or shControl. These cells were seeded in 12-well plates for 24 h. The cells were then transfected with p53*-*luc or pRL-TK vector containing Renilla luciferase (Promega, Madison, WI, USA) using Lipofectamine 2000 (Invitrogen, Carlsbad, CA, USA). Luciferase activities were measured 36 h post-transfection with a Dual-luciferase reporter kit (Promega, Madison, WI, USA) according to the manufacturer's protocol. Renilla luciferase activity was used to normalize Firefly luciferase activity.

### Patient samples and immunohistochemistry

A total of 123 paraffin-embedded tissue samples were collected from HCC patients at the Sun Yat-sen University Cancer Center from 2008 to 2012.All of the patients underwent curative surgical resection and none of them received chemotherapy, radiotherapy or targeted therapy before the surgery. Consecutive 3 µm thick sections were excised from the paraffin-embedded tissue samples for immunohistochemistry according to standard protocols. Briefly, the sections were deparaffinized, rehydrated and washed. Samples were microwaved and antigen retrieval was completed in Tris-EDTA. Further, endogenous peroxidase and non-specific binding were blocked with 0.3% H_2_O_2_ and goat serum, respectively. The specimens were then incubated with 1º antibodies against human BRD7 (1:100, Cell Signaling Technology) or WTp53 (p53 (DO-1) Mouse mAb #18032, 1; 200; Cell Signaling Technology) overnight at 4 ºC. The slides were serially washed, incubated with HRP-conjugated 2º antibodies, visualized by 3, 3'-diaminobenzidine tetrahydrochloride and counterstained with hematoxylin followed by dehydration and evaluation. BRD7 expression was assessed as previously described 12. Briefy, both the percentage of positively stained tumor cells and the staining intensity among the total tumor tissue were scored as “0” (no staining and <5%, respectively), “1” (weakly stained and 5%-25%, respectively),“2”(moderately stained and 25%-50%, respectively) or “3”(well stained and >50%, respectively). Multiplication of the intensity and percent positivity was classified as the final immunostaining score (ranging from 0 to 9). The BRD7 expression was determined as low (0-3) or high (4-9) according to the final immunostaining score. For evaluating p53 expression, the immunostaining score was quantified as a p53 index (the number of p53 positive cells per every 1000 counted cells) and the ImmunoHistoChemistry (IHC) score was identified as 0 (p53 index<5), 1 (p53 index 5-50), 2 (p53 index 50-100), or 3 (p53 index >100). The samples were further divided into low p53 expression (0-1) and high p53 expression (2-3). All immunostained specimens were evaluated by two separate researchers who were blinded to the clinical outcome of the patients.

### qRT-PCR and western blot analysis

The real-time quantitative polymerase chain reaction (qRT-PCR) and western blot analysis were performed as previously described 12. In brief, total RNA was isolated from cell lines using TRIzol reagent (Invitrogen, CA, USA) according the manufacturer's protocol. cDNA that had been synthesized from RNA was subjected to real-time quantitative polymerase chain reaction (qRT-PCR) to evaluate the differential expression of p53 mRNA using a SYBR Green Master Mix (Invitrogen) and the ABI Prism 7900HT Sequence Detection System. The primer sequences for WTp53 were as follows, 5′-AACAACACCAGCTCCTCTCC -3′ (forward) and 5′-CTCATTCAGCTCTCGGAACA -3′ (reverse). The relative expression of p53 mRNA was analyzed using the 2^-ΔΔCT^ method. The GAPDH mRNA expression was used for normalization and its primer sequences was 5′-CTCCTCCTGTTCGACAGTCAGC-3′ (forward) and 5′-CCCAATACGACCAAATCCGTT-3′ (reverse). For western blot analysis, proteins were extracted from cell lines using RIPA lysis buffer, separated by 12% sodium dodecyl sulfate (SDS)-polyacrylamide gel electrophoresis, and transferred to a polyvinylidene fluoride membrane (PVDF). The membranes were blocked with 5% non-fat milk, and incubated with monoclonal antibodies against BRD7, Bax, Bcl2 ,cyclin D1, GAPDH (1:500, Proteintach), p53, p21, Akt, p-Akt, p-PTEN, and β-catenin (1:1000; Cell Signaling Technology), overnight at 4 ºC. Membranes were then washed with TBST and incubated with horseradish peroxidase-conjugated 2º antibody. Immunoreactive signals were detected using an enhanced chemiluminescence system (Cell Signaling Technology).

### Cell proliferation and colony formation assays

The cell proliferation and colony formation assays were performed as previously described 12. In Brief, cell lines were seeded in 96-well plates at a density of 1,500 cells/well, and the proliferation rates were evaluated using an MTS cell proliferation kit (Promega, USA) according to manufacturer's protocol. To analyze colony formation the cell lines were seeded into 6-well plates at a density of 1,000 cells/well and incubated in a humidified chamber for 10 days. The cells were then washed and fixed. Positive colonies containing more than 50 cells were counted, and the colony forming efficiency was calculated as (colony number/plated cell number) × 100. All experiments were performed in triplicate.

### Cell migration and invasion assays

The cell-migration and invasion assays were conducted using 24-well transwell chamber system (8.0 µm pore size, Corning). For cell-migration, a total of 1× 10^5^ cells resuspended in 100 µl of media was added to the upper chamber (without Matrigel). For cell-invasion, 2 × 10^5^ cells were plated in the upper chamber pre-coated with 0.5 mg/ml Matrigel. After incubation at 37 ºC for 24 h or 48 h, the cells that had migrated to or invaded the lower chamber were harvested and counted using a hemocytometer.

### Cell cycle assay

Bel7402 and HepG2 cells were collected and washed in ice-cold PBS following 72 h transfection. The cells were then fixed in ice-cold 75% ethanol at -20 ºC overnight, after which they were rinsed and resuspended in PBS and incubated with RNase at 37 ºC for 30 min. The cells were then stained with PI for 1 h at 4 ºC in the dark. Flow cytometry was subsequently completed to analyze the cell cycle distribution. All experiments were performed in triplicate.

### Xenograft Transplantation

Four-week-old female BALB/c nude mice were obtained from Vital River Laboratory Animal Technology Co., Ltd. (Beijing, China). The mice were randomly divided into 6 groups and subcutaneously injected with 5×10^6^ Bel7402/Contorl-1, Bel7402/ BRD7-1, Bel7402/ BRD7+shp53, HepG2 /Contorl-1, HepG2/shBRD7, HepG2 /shBRD7+p53, respectively. The tumor growth was assessed every 4 days by measuring the tumor volume calculated as (length × width^2^)/2. All mice were euthanized after 24 days. The tumor grafts were excised, harvested, photographed and weighed. All animal experiments were performed according to the institutional ethical guidelines for animal experiments and the Guide for the Care and Use of Laboratory Animals (NIH publication Nos. 80-23, revised 1996).

### Patient follow-up

All patients in this study received regular follow-up from the outpatient department or from the follow-up center at Sun Yat-sen University Cancer Center. Overall survival (OS), which was defined as the time from the date of surgery to the date of death or the last follow-up, was used to measure prognosis for the patients.

### Statistical analysis

The relationship between tumor BRD7 and p53 expression was determined using chi-square analysis and Pearson's correlation coefficients. The Kaplan-Meier method was performed for survival curves, and statistical significance was calculated using the log-rank test. Mann-Whitney U test, Student's t-test or one-way ANOVA were used to compare the differences between subgroups. The results are shown as the mean ± SD. All statistical analyses were conducted using SPSS version 20.0 or GraphPad Prism 5 software. A two-tailed p value<0.05 was considered statistically significant.

## Results

### ChIP-sequencing identifies BRD7 binding targets in HCC cell lines

**Figure 1:**
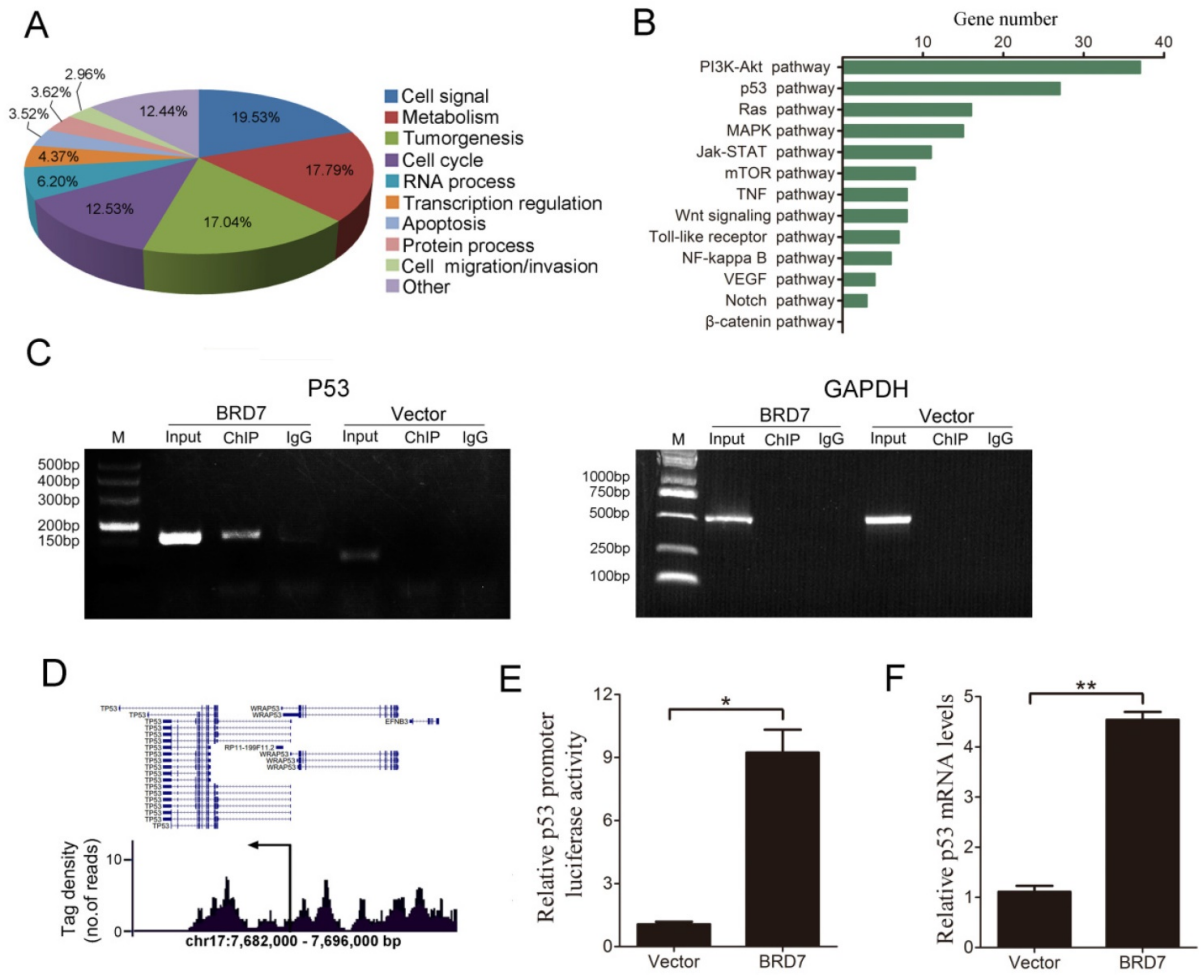
**Defining genome-wide BRD7 binding genes by ChIP-sequencing in Bel7402 cells transfected with BRD7. (A)** Functional annotation of BRD7-associated genes based on enriched GO terms. **(B)** The most significant “pathways in cancer” enriched by BRD7 target genes are presented, based on KEGG pathway analysis. **(C)** ChIP-PCR analysis of the binding of BRD7 to p53 gene in Bel7402 cells transfected with BRD7 or Vector. Primers were designed against the peak interval sequence, and GAPDH served as the negative control. **(D)** Representative tag density profiles for BRD7-bound regions located in p53, and their proximity to the transcriptional initiation site (arrow).** (E)** The potential p53 promoter activity in Bel7402 cells transfected with BRD7 by the dual-luciferase reporter assay, normalized to the pRL-TK vector containing Renilla luciferase. **(F)** Fold changes in p53 mRNA levels in Bel7402 cells transfected with BRD7 compared to Bel7402 cells transfected with Vector. * p<0.05, ** p<0.01.

BRD7 has been shown to act as a transcriptional regulator 20-22, therefore, we conducted ChIP-seq analysis to characterize genome-wide BRD7 binding sites using BRD7-overexpressing HCC cells (HepG2 and Bel7402). Our results indicate that significant enrichment of BRD7-binding sites was identified using stringent statistical criteria (fold change of more than 1.5, p value<0.01). Based on enriched Gene Ontology (GO) terms, most of the genes bound by BRD7 were involved in cell signaling, metabolism, tumorigenesis, cell cycle progression, RNA processing, transcription regulation, apoptosis, and cell migration/invasion, among others (Figure 1A and Figure S1A). Furthermore, KEGG Pathway analysis revealed that a large number of BRD7 binding targets were involved in “pathways in cancer” which were found to be enriched in genes associated with the p53 and PI3K-Akt pathways (Figure 1B and Figure S1B). Alternatively, from the TCGA data, BRD7 was reported to be involved in the PI3K-Akt pathway in HCC cells, but not in the p53 pathway (Figure S2).We therefore explored the relationship between BRD7 and the p53 pathway. ChIP-PCR analysis results showed that BRD7 can directly bind to p53 DNA in BRD7-overexpressing Bel7402 cells and HepG2 cells transfected with control siRNA (siControl) (Figure 1C and Figure S1C). Analysis of the tag density distribution using MACS2 software revealed that most BRD7-bound regions were located at the upstream regulatory region of p53 transcriptional initiation site (Figure 1D and Figure S1D). In order to determine whether BRD7 bound directly to the promoter regions of p53, we performed dual-luciferase reporter assays in Bel7402/BRD7 cells and compared the results with those of control cells. As shown in Figure 1E, the luciferase activity of the p53 promoter was found to be significantly increased in BRD7-overexpressing cells. In addition, we verified, by qRT-PCR analysis, that p53 mRNA expression was indeed up-regulated by BRD7 (Figure 1F). Opposite results were obtained when verifying the p53 promoter luciferase reporter and p53 mRNA expression using BRD7 knockdown in HepG2 and Bel7402 cells (Figure S1E and S1F, and Figure S3). These results suggest that BRD7 may act as a transcription factor that contributes to activating p53 pathway in HCC cells.

### BRD7 expression was positively correlated with p53 protein in HCC patients

**Figure 2:**
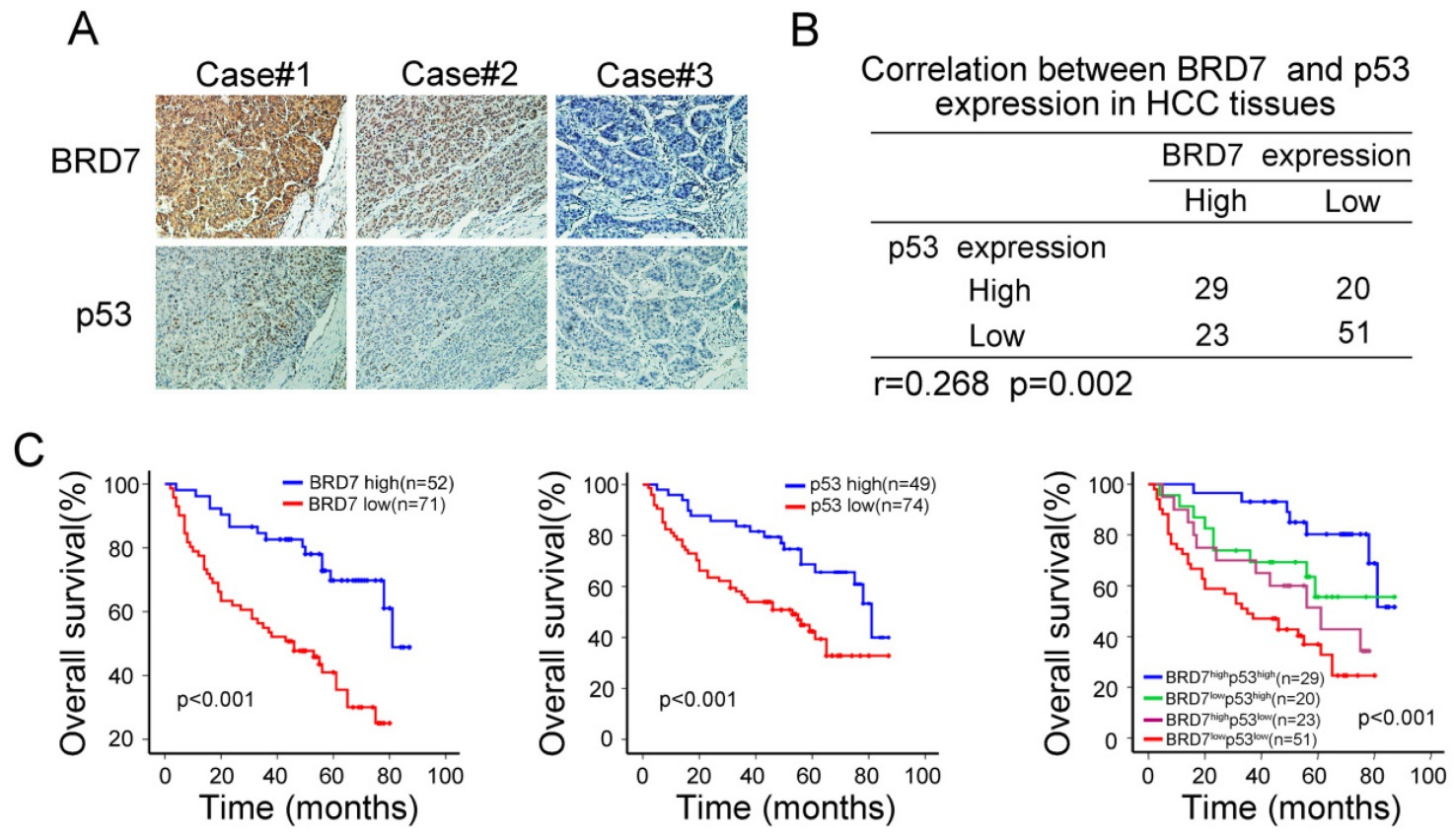
**Immunohistochemical analysis of the relationship between BRD7 p53 protein expression in HCC patients. (A)** Immunohistochemical staining of BRD7 and WTp53 in the same tumor tissues. Original magnification ×200.** (B)** Statistical analysis of the correlation between BRD7 and p53 expression in HCC tissues.** (C)** Overall survival curves for the HCC patients.

The observed transcriptional regulation of p53 by BRD7 led us to validate the association between the expression of BRD7 and p53 protein levels. As mutations in p53 are common oncogenic driver in HCC, We used WTp53 antibody to detect p53 expression levels by immunohistochemical analysis in serial HCC tissue sections. We observed an increase in p53 expression in tumor tissues that highly expressed BRD7, while decreased p53 expression was related to lower BRD7 expression in the same tumor tissue (Figure 2A). Correlation analysis further demonstrated that the expression level of BRD7 positively correlated with the protein level of p53 in HCC patients (n =123, r =0.268, P=0.002, Figure 2B). Further, we classified all HCC patients into four groups based on BRD7 and p53 expression. Survival analysis suggested that both BRD7^high^ and p53^high^ patients exhibited a better overall survival compared to BRD7^low^ and p53^low^ patients, respectively. In addition, simultaneously, patients with BRD7^high^ and p53^high^ have the most favorable prognosis in HCC patients (Figure 2C). These findings indicated that both BRD7 and p53 may be involved in HCC progression.

### p53 is involved in BRD7-mediated inhibition of cell proliferation, migration/ invasion , and cell cycle in HCC cells

**Figure 3:**
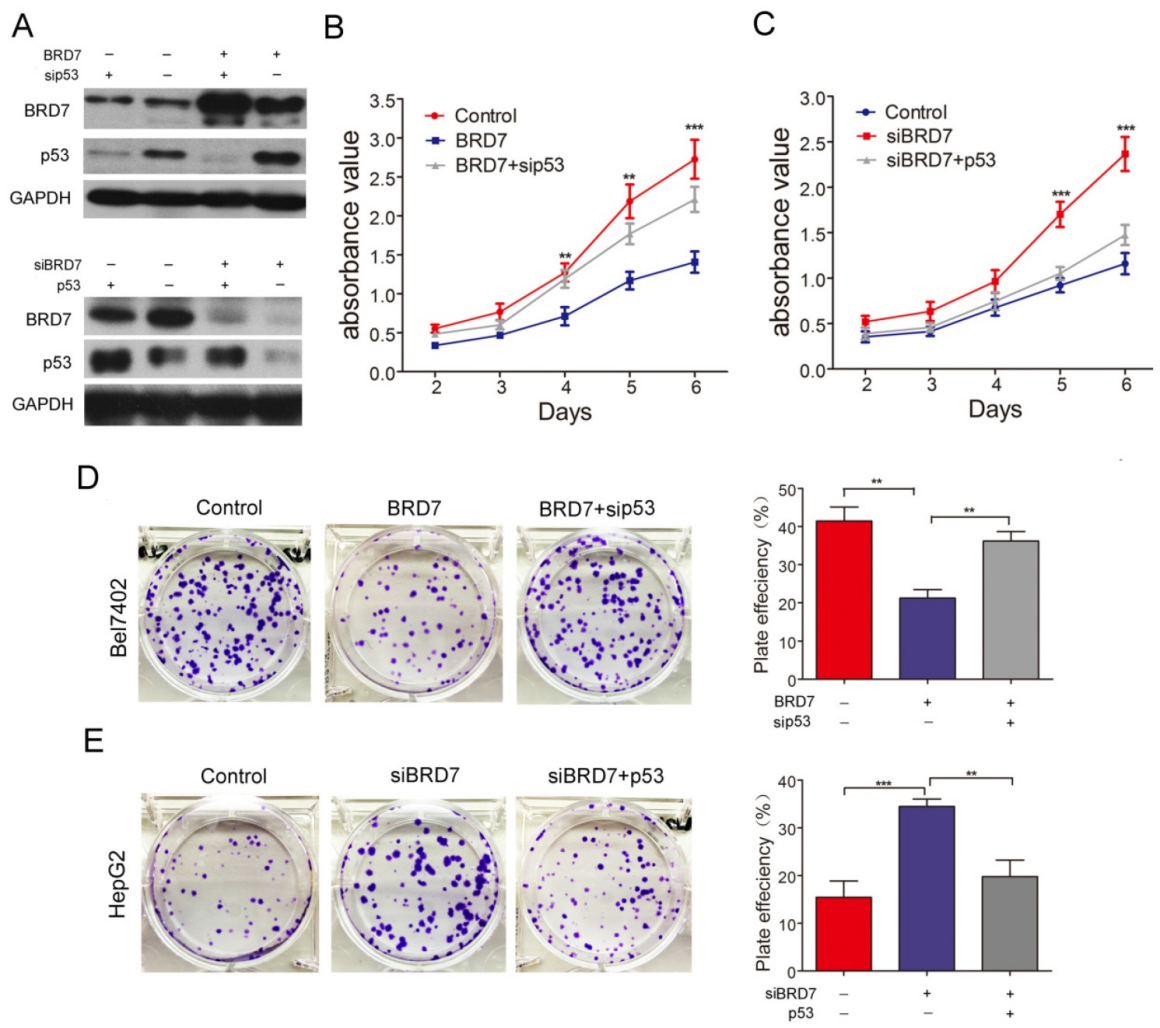
**Effect of p53 expression on BRD7 inhibition of *in vitro* cell growth in HCC cells. (A)** Western blotting analysis of Bel7402 cells co-transfected with BRD7 and/or sip53 (Up) and HepG2 cells co-transfected with siBRD7 and/or p53 (Down). **(B)** MTT assays of Bel7402 cells co-transfected with vector and siControl, BRD7 and siControl, or BRD7 and sip53, respectively.** (C)** MTT assays of HepG2 cells co-transfected with siControl and vector, siBRD7 and vector, or siBRD7 and p53, respectively. **(D)** Colony-formation assays and quantification of colony number/well for each group of the Bel7402 cells. **(E)** Colony-formation assays and quantification of colony number/well for each group of the HepG2 cells. All experiments were performed in triplicate. The mean ± SD of the foci for each group are shown. ** p<0.01, *** p<0.001.

**Figure 4:**
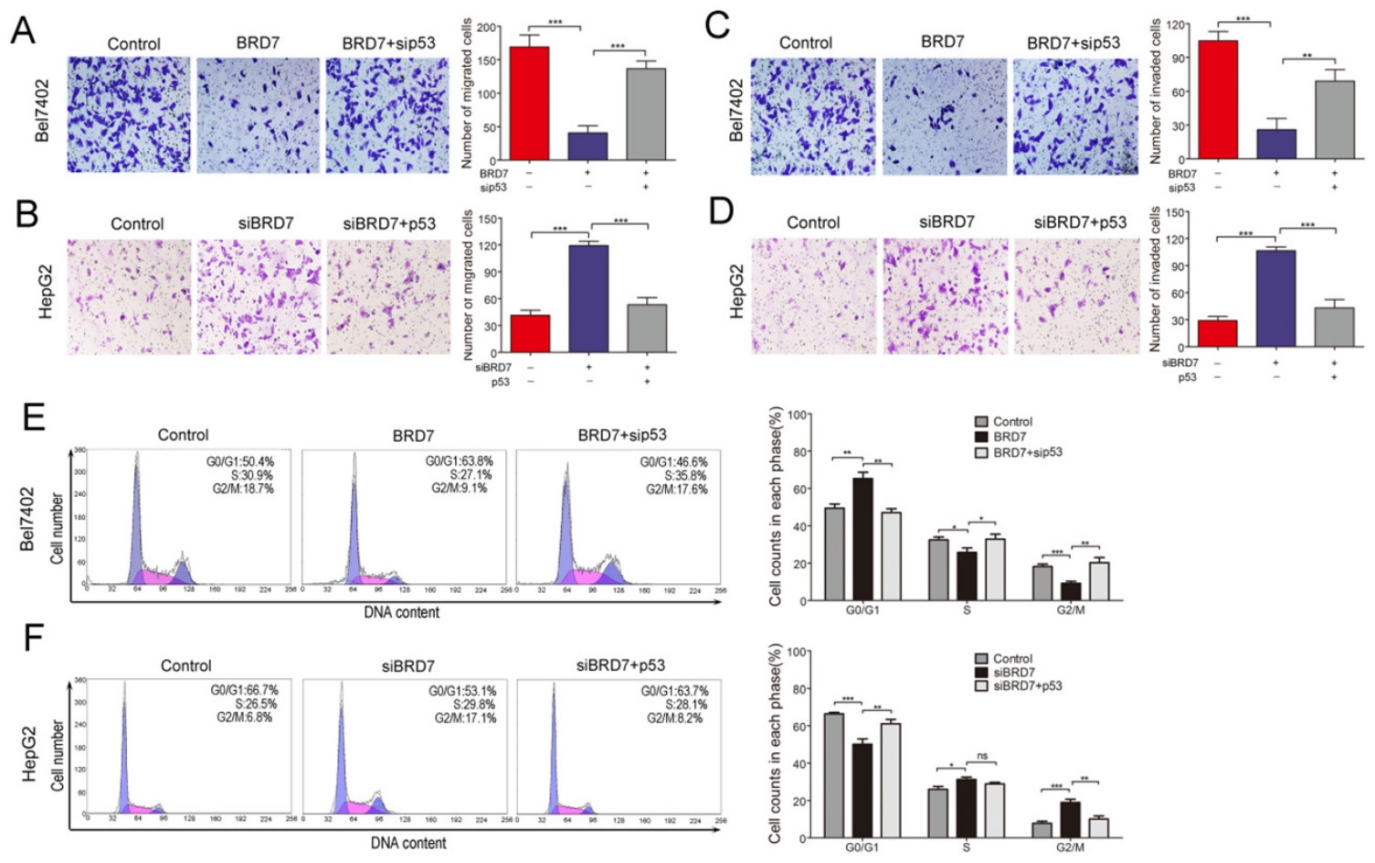
** Effect of p53 expression on BRD7 inhibition of migration, invasiveness and cell cycle in HCC cells. (A)** Transwell migration assay for Bel7402 cells co-transfected with vector and siControl, BRD7 and siControl, or BRD7 and sip53, respectively.** (B)** Transwell migration assay for HepG2 cells co-transfected with siControl and vector, siBRD7 and vector, or siBRD7 and p53, respectively.** (C)** Matrigel invasion assay for each group of the Bel7402 cells. **(D)** Matrigel invasion assay for each group of the HepG2 cells. Images are shown in the left panel at 100× magnification; the quantification of 6 randomly selected fields is shown in the right panel. **(E)** Cell cycle assay of Bel7402 cells co-transfected with vector and siControl, BRD7 and siControl, or BRD7 and sip53, respectively.** (F)** Cell cycle assay of HepG2 cells co-transfected with siControl and vector, siBRD7 and vector, or siBRD7 and p53, respectively. All experiments were performed in triplicate. The mean ± SD of the foci for each group are shown. * p<0.05, ** p<0.01, *** p<0.001.

BRD7, as a potential tumor suppressor, may suppress HCC tumorigenicity^12^. BRD7 has demonstrated the ability to enhance p53 expression by transcriptional regulation. Therefore, we investigated whether the suppressive effect of BRD7 on various cell functions is associated with p53 activity in HCC cells. The various rescue assays were carried out by transfecting Bel7402 cells with BRD7-overexpression recombinant lentiviruses (BRD7) and/or p53siRNA (sip53) and HepG2 cells with siBRD7 and/or p53-overexpression plasmid (p53), respectively. The knockdown or restoration of BRD7 and p53 was confirmed via western blot analysis (Figure 3A). We performed cell proliferation and colony-forming assays to determine the impact of p53 knockdown or restoration on the growth of HCC cells. As expected, downregulation of p53 expression in Bel7402 cells overexpressing BRD7 (BRD7+ sip53) caused significantly increased cell proliferation and colony-formation compared to BRD7-overexpressing control cells (BRD7) (Figure 3B and 3D), whereas restoring the p53 level in HepG2 cells that were co-transfected with siBRD7 (siBRD7+ p53) continued to decrease the levels of cell proliferation and colony-formation compared with BRD7-knockdown control cells (siBRD7) (Figure 3C and 3E). In addition, we analyzed the effect of p53 knockdown or restoration on cell migration and invasiveness. The results showed that the migration ability and invasiveness of cells were remarkably enhanced in BRD7-overexpressing Bel7402 cells co-transfected with sip53 when compared to BRD7-overexpressing control cells (Figure 4A and 4C), while knockdown of BRD7 did not impact cell migration or invasion following restoration of p53 expression in HepG2 cells (Figure 4B and 4D). Furthermore, we evaluated the possible effects of p53 expression on BRD7-mediated cell-cycle arrest.

We found that overexpression of BRD7 did not increase the percentage of Bel7402 cells in G0/G1 phase and did not decrease the S-phase or G2/M population in the absence of p53 expression (Figures 4E). However, restoring p53 expression caused a significant increase in the G0/G1 cell fractions, and a subsequent decrease in the G2/M population within HepG2 cells co-transfected siBRD7 compared to those cells with BRD7-knockdown only (Figures 4F). All these findings indicate that the *in vitro* BRD7-mediated tumor suppressive effect on cell growth, cell migration and invasion, and cell-cycle progression requires p53 activity in HCC cells.

### The ability of BRD7 to inhibit tumor growth* in vivo* is dependent on the p53 tumor suppressor protein

**Figure 5:**
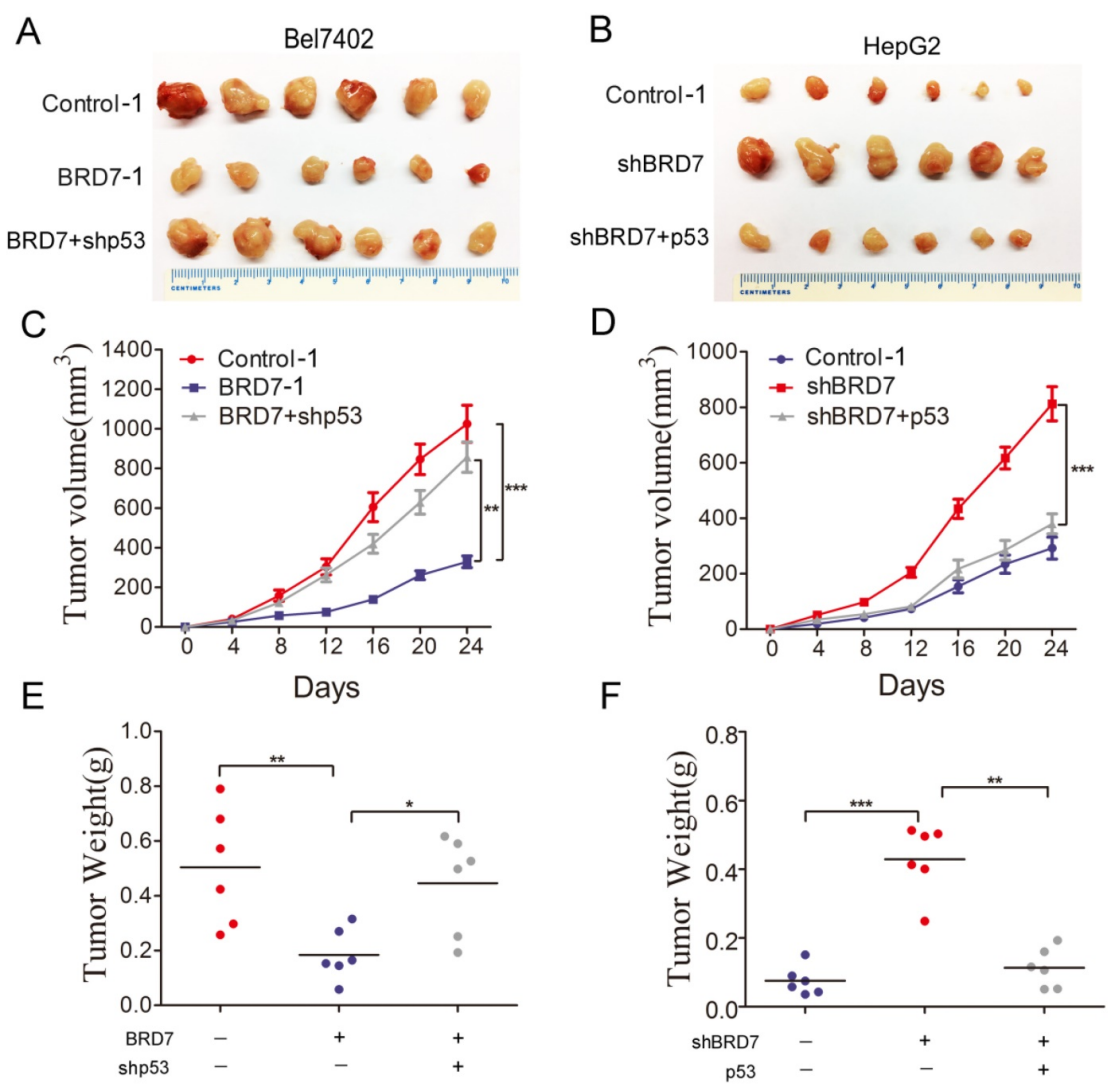
** Effect of p53 expression on the tumor suppressive role of BRD7 *in vivo* (A)** Tumor images from BALB/c nude mice (n=6) for Bel7402 cells co-transfected with vector and shControl, BRD7 and shControl, or BRD7 and shp53, respectively.** (B)** Tumor images from BALB/c nude mice (n=6) for HepG2 cells co-transfected with shControl and vector, shBRD7 and vector, or shBRD7 and p53, respectively.** (C)** Tumor growth curves of these Bel7402 cells xenograft mouse model.** (D)** Tumor growth curves of these HepG2 cells xenograft mouse model.** (E)** The tumor weights of each group of Bel7402 cells are shown.** (F)** The tumor weights of each group of HepG2 cells are shown. The data are presented as the means ± SD. * P<0.05, ** P<0.01, *** P<0.001.

To validate our *in vitro* findings, we established *in vivo* experiments with a xenograft BALB/c nude mouse model. Bel7402/Contorl-1, Bel7402/ BRD7-1, and Bel7402/BRD7+shp53 were injected subcutaneously into nude mice, respectively. The results revealed that p53 knockdown in Bel7402 cells overexpressing BRD7 significantly promoted xenograft tumor growth compared to those cells overexpressing BRD7 only(Figure 5A). At the end of the observation period, the mean tumor volume and weight of the xenograft tumors in p53 knockdown mice were remarkably increased compared to that of the BRD7-overexpression only group (Figure 5C and 5E). We also subcutaneously injected HepG2/Contorl-1, HepG2/shBRD7, HepG2/shBRD7+p53 into nude mice, respectively. We found that the xenograft tumor growth was significantly delayed in HepG2 cells transfected with shBRD7 following p53-overexpression compared to BRD7-knockdown only group (Figure 5B). Accordingly, the mean tumor volume and weight at the end of observation were markedly lower in the xenograft tumors with p53 restoration compared to those in the BRD7-knockdown only group (Figure 5D and 5F). Corresponding with our* in vitro* findings, these *in vivo* results suggested that BRD7-mediated antitumor effects in HCC occurred in part due to its positive transcriptional regulation of p53.

### BRD7 positively regulates the expression of specific proteins in the p53 pathway in HCC

**Figure 6:**
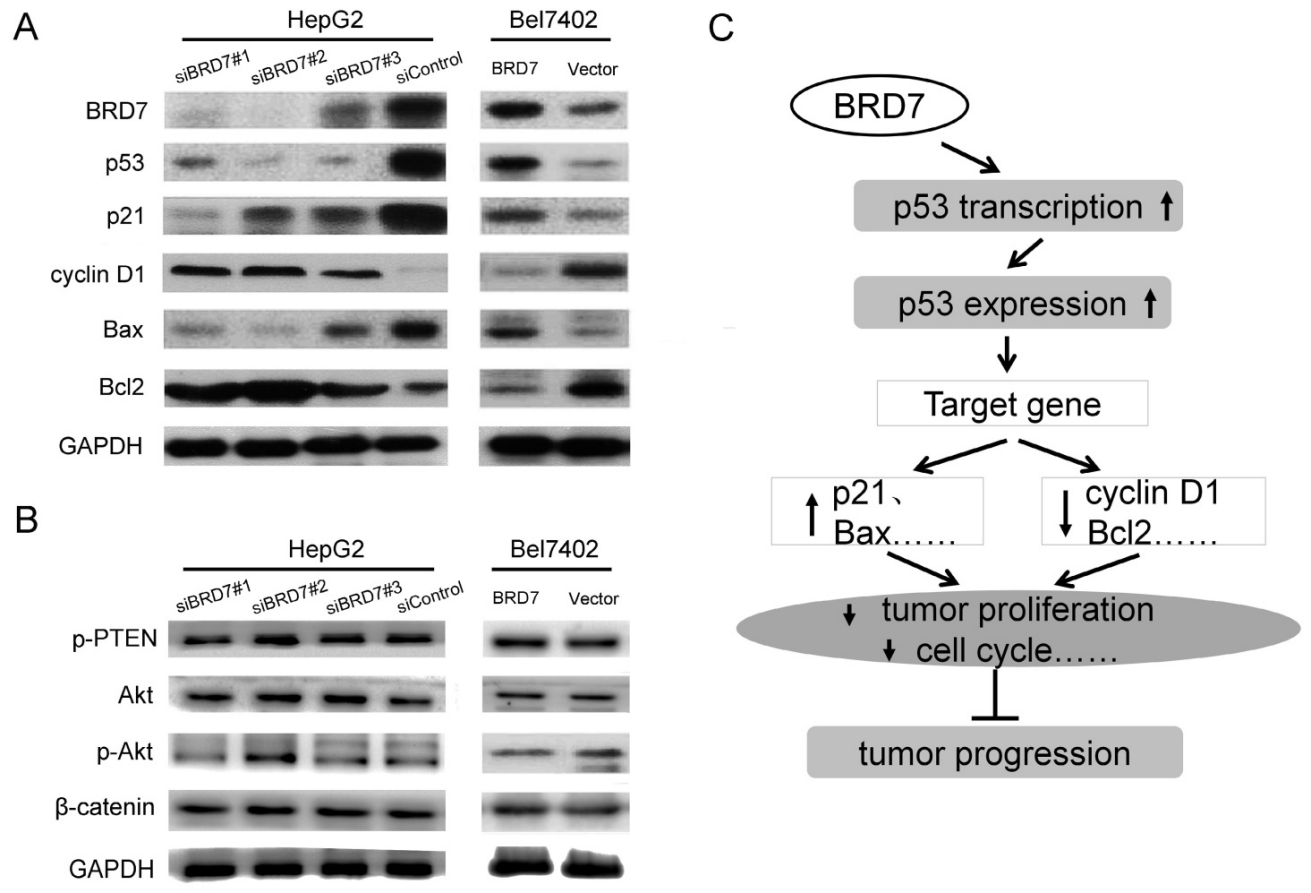
** BRD7 regulates the p53 pathway in HCC cells. (A)** Western blot analysis for proteins involved in p53 signaling in BRD7-knockdown HepG2 cells (Left) and in BRD7-overexpressed Bel7402 cells (Right). **(B)** Western blot analysis for proteins involved in the PI3K signaling pathway in these HCC cells. **(C)** A schematic map for the tumor suppressive mechanism of BRD7 in HCC that occurs by transcriptionally regulating the p53 pathway.

It is well known that the p53 pathway plays a critical role in regulating cellular behavior in various cancer cells 23,24. In this signaling, p53 regulates cell survival and cell-cycle progression through transcriptional regulation of its target genes including p21, Bax, Bcl2, and cyclin D1 25,26. Our findings clearly demonstrated that BRD7 upregulates the p53 transcriptionally, we therefore, wanted to determine whether BRD7 is involved in regulation of other proteins related to p53 signaling in HCC using BRD7-overexpressing cells. Western blot analysis revealed that overexpression of BRD7 in Bel7402 cells led to an increase in the protein levels of p53, p21, and Bax, and a decrease in the expression of cyclin D1 and Bcl2 protein (Figure 6A). We also generated BRD7-knockdown cells. As expected, reduction in the expression of BRD7 within HepG2 cells significantly associated with downregulation of p53, p21, and Bax expression, and with upregulation of cyclin D1 and Bcl2 expression (Figure 6A). Alternatively, although BRD7 was found to be involved in the PI3K pathway by ChIP-seq analysis (Figure 1C and Figure S1C), overexpression or knockdown of BRD7 in HCC cells did not significantly affect the protein levels of PTEN, Akt, and p-Akt which are central components in the PI3K pathway (Figure 6B). These results confirmed that BRD7 may function as a transcription factor that inhibits tumor progression by directly targeting p53, specifically by enhancing p53 expression and promoting the activation of the p53 pathway (Figure 6C).

## Discussion

As a subunit of the PBAF-specific SWI/SNF chromatin-remodeling complex, BRD7 has been found to interact with other proteins and modify their activity by regulating acetylation of histones 22,27. Recent evidence has demonstrated that BRD7 also serves as a tumor suppressor by regulating tumor-related signaling pathways involved in cell growth, mobility and cell cycling 28,29. Further, consistent with multiple studies demonstrating a critical role for BRD7 in the progression of multiple cancers 11,30, our previous study confirmed that BRD7 acts as an anti-tumor factor in HCC progression^12^. However, the molecular mechanism underlying this inhibitory activity seems to differ between tumor types. For example, Park et al*.*revealed that BRD7 suppresses epithelial ovarian cancers independent of p53 activity via negative regulation of the β-catenin pathway 31. In another study, BRD7 was found to inhibit cell proliferation in breast cancer tumors by activating a Bcl2-antagonist/killer protein 32. Moreover, Chiu et al. described a tumor suppressor role for BRD7 that functions by interacting with p85α and negatively regulating PI3K activity in cervical cancer cells 16. Our current study is the first, to our knowledge, to demonstrate that BRD7 may inhibit tumor progression in HCC through positively regulating the p53 pathway. These divergent findings suggest that the mechanism by which BRD7 exerts its antitumor effect is complex and variable and that it is highly reliant on the specific tumor type.

Recent evidence has shown that BRD7 regulates the transcriptional activity of its target genes and of downstream signaling pathways, such as p53, BRCA1, and BRD2 15,20,33. However, given the multiple signaling pathways involved in the tumor suppressive effect of BRD7, the target genes and functional networks associated with BRD7 have not yet been clearly explored in the context of HCC. Therefore, in this study, we performed chromatin immunoprecipitation, followed by sequencing (ChIP-seq) analysis to investigate the regulating network of BRD7 and its effect on downstream genes in HCC. ChIP-seq analysis is one of the most commonly used techniques for evaluating the binding sites and functional mechanism of transcriptional factors. It is capable of generating large quantities of data, which can be used to elucidate gene regulatory networks and characterize the interplay between the transcriptome and epigenome 34,35. Our ChIP-seq results describe the target genes directly bound to BRD7 in human HCC. Following pathway analysis of the BRD7-binding targets, we found that many of functional networks associated with BRD7 were involved in cell cycling and cell survival. These results are consistent with previously described functions for BRD7 in apoptosis and cell cycle progression 13,14,36. In addition, a recent study conducted by Xu et al. reported on an integrative analysis of genome-wide chromatin occupancy for BRD7 by ChIP-seq and digital gene expression (DGE) analysis in kidney epithelial cells and cervical cancer cells. They constructed a regulating network of genes that are downstream from BRD7, and validated BIRC2, BIRC3, and NOTCH1 genes as direct, functional BRD7 targets, which are all involved in cell cycling and apoptotic pathways 37.

Among the “pathways in cancer” that were identified as enriched in the BRD7-binding peaks, we specifically demonstrate that the p53 signaling pathway, which is well known to control cell cycling and proliferation of cancer cells, is functionally regulated by BRD7 in HCC cells. We found that BRD7 enhanced the transcription level of p53by directly binding to upstream regulatory regions of the p53 transcriptional initiation site, thereby enhancing the activity of the p53 promoter. Additionally, as an essential mediator of the p53 signaling pathway, the p53 tumor suppressor protein has an integral role in regulating cellular behavior in cancer cells through transcriptionally regulating target genes in signaling pathways associated with critical cellular functions 38. In HCC, it is well established that p53 can restrict malignant progression by initiating cell cycle arrest, apoptosis, and senescence in response to cellular stress 39. Alternatively, loss of p53 activity has been shown to cause enhanced transformation of adjacent epithelial cells into hepatocellular carcinoma 40.

Therefore, given the critical role that p53 has in limiting tumorigenesis and the transcriptional regulation of p53 by BRD7, we investigated whether the molecular mechanism by which BRD7 suppress HCC tumorigenesis is directly associated with the activity of the p53 signaling pathway. As expected, our results showed that the expression of BRD7 and p53 is significantly positively correlated in HCC tissues, and that both proteins were associated with favorable prognosis in HCC patients. In addition, we determined that BRD7-mediated tumor suppressive effects were significantly attenuated by downregulation of p53 expression in HCC cells. These results suggest that the tumor suppressive functions of BRD7 in HCC may involve a p53-dependent signaling pathway. Indeed, changes in BRD7 expression markedly altered the expression of p53 signaling pathway-related proteins including p21, Bax, Bcl2, and cyclin D1, indicating that BRD7 may positively regulate the activation of the p53 pathway. Consistent with our present findings, a previous study has shown that BRD7 regulates the p53 pathway by binding acetylated histones, thereby facilitating sustained and proper acetylation of histones surrounding p53-binding sites during transcription, which is necessary for transcriptional activation to occur in a subset of p53 target genes 36. Another study demonstrated, however, that BRD7 inhibited cancer cell survival, apoptosis, and migration/invasion independent of the p53 signaling pathway, but through negatively regulating β-catenin pathways 31. Indeed, besides its crucial role in the p53 pathway, BRD7 may also regulate the transcription of other genes not associated with the p53 pathway, that are involved in regulation of cellular behavior. Recently it was demonstrated, in overexpression studies, that BRD7 transcriptionally regulates components of the Rb/E2F and Ras/MEK/ERK pathways 13,14. Moreover, BRD7 has also been identified as a p85a-interacting protein that attenuates PI3K signaling and maintains the homeostasis of cell growth in cancer cells 16. Nevertheless, we found that changing the expression levels of BRD7 did not alter the activity of essential components (PTEN and Akt) in the PI3K signaling pathway, although BRD7 was shown to be associated with the PI3K pathway through our ChIP-seq analysis.

In summary, our study is the first to confirm the genome-wide chromatin occupancy and the regulating gene network of BRD7 using ChIP-seq analysis for elucidating the biological function of BRD7 in HCC. Moreover, we identified the p53 pathway as a target of BRD7. We also demonstrated that BRD7 activates p53 signaling by transcriptionally upregulating p53 protein expression, which contributes to inhibition of cellular growth, motility and cell-cycling in HCC cells. These findings suggest that BRD7 acts as a tumor suppressor in HCC by positively regulating p53 pathway activity. Establishing this molecular mechanism of BRD7 may provide promising diagnostic and therapeutic strategies for HCC. However, the mechanism by which BRD7 transcriptionally regulates the p53 pathway remains to be elucidated. On the other hand, p53 seems to exerts a negative feedback regulation on BRD7 expression in this study (Figure 3A,down). Indeed, as a transcription factor,p53 can form an auto-regulatory feedback loop with its regulator such as MDM2 41. Nevertheless, how p53 in turn regulate BRD7 expression in HCC remains unclear,which should be further investigated.

## Supplementary Material

Supplementary figures and table.

## References

[1] Bray F, Ferlay J, Soerjomataram I (2018). Global cancer statistics 2018: GLOBOCAN estimates of incidence and mortality worldwide for 36 cancers in 185 countries. CA Cancer J Clin.

[2] Bruix J, Gores GJ, Mazzaferro V (2014). Hepatocellular carcinoma: clinical frontiers and perspectives. Gut.

[3] Abou-Alfa GK, Meyer T, Cheng AL (2018). Cabozantinib in Patients with Advanced and Progressing Hepatocellular Carcinoma. N Engl J Med.

[4] El-Khoueiry AB, Sangro B, Yau T (2017). Nivolumab in patients with advanced hepatocellular carcinoma (CheckMate 040): an open-label, non-comparative, phase 1/2 dose escalation and expansion trial. Lancet.

[5] Llovet JM, Montal R, Sia D (2018). Molecular therapies and precision medicine for hepatocellular carcinoma. Nat Rev Clin Oncol.

[6] Forner A, Reig M, Bruix J (2018). Hepatocellular carcinoma. Lancet.

[7] Peng C, Liang SP, Tan C (2003). Researching a novel NPC-related candidate suppressor gene BRD7 by two-dimensional gel electrophoresis and MALDI-TOF-MS. Sheng Wu Hua Xue Yu Sheng Wu Wu Li Xue Bao (Shanghai).

[8] Tae S, Karkhanis V, Velasco K (2011). Bromodomain protein 7 interacts with PRMT5 and PRC2, and is involved in transcriptional repression of their target genes. Nucleic Acids Res.

[9] Wang H, Zhao R, Guo C (2016). Knockout of BRD7 results in impaired spermatogenesis and male infertility. Sci Rep.

[10] Niu W, Luo Y, Wang X (2018). BRD7 inhibits the Warburg effect and tumor progression through inactivation of HIF1alpha/LDHA axis in breast cancer. Cell Death Dis.

[11] Yu X, Li Z, Shen J (2016). BRD7: a novel tumor suppressor gene in different cancers. Am J Transl Res.

[12] Chen CL, Wang Y, Pan QZ (2016). Bromodomain-containing protein 7 (BRD7) as a potential tumor suppressor in hepatocellular carcinoma. Oncotarget.

[13] Zhou J, Ma J, Zhang BC (2004). BRD7, a novel bromodomain gene, inhibits Gl-S progression by transcriptionally regulating some important molecules involved in ras/MEK/ERK and Rb/E2F pathways. J Cell Physiol.

[14] Peng C, Liu HY, Zhou M (2007). BRD7 suppresses the growth of Nasopharyngeal Carcinoma cells (HNE1) through negatively regulating beta-catenin and ERK pathways. Mol Cell Biochem.

[15] Harte MT, O'Brien GJ, Ryan NM (2010). BRD7, a subunit of SWI/SNF complexes, binds directly to BRCA1 and regulates BRCA1-dependent transcription. Cancer Res.

[16] Chiu YH, Lee JY, Cantley LC (2014). BRD7, a tumor suppressor, interacts with p85alpha and regulates PI3K activity. Mol Cell.

[17] Liu Y, Zhao R, Wei Y (2018). BRD7 expression and c-Myc activation forms a double-negative feedback loop that controls the cell proliferation and tumor growth of nasopharyngeal carcinoma by targeting oncogenic miR-141. J Exp Clin Cancer Res.

[18] Liu T, Zhao M, Liu J (2017). Tumor suppressor bromodomain-containing protein 7 cooperates with Smads to promote transforming growth factor-β responses. Oncogene.

[19] Zhao R, Liu Y, Wang H (2017). BRD7 plays an anti-inflammatory role during early acute inflammation by inhibiting activation of the NF-кB signaling pathway. Cell Mol Immunol.

[20] Burrows AE, Smogorzewska A, Elledge SJ (2010). Polybromo-associated BRG1-associated factor components BRD7 and BAF180 are critical regulators of p53 required for induction of replicative senescence. Proc Natl Acad Sci U S A.

[21] Penkert J, Schlegelberger B, Steinemann D (2012). No evidence for breast cancer susceptibility associated with variants of BRD7, a component of p53 and BRCA1 pathways. Fam Cancer.

[22] Kaeser MD, Aslanian A, Dong MQ (2008). BRD7, a novel PBAF-specific SWI/SNF subunit, is required for target gene activation and repression in embryonic stem cells. J Biol Chem.

[23] Wang Z, Sun Y (2010). Targeting p53 for Novel Anticancer Therapy. Transl Oncol.

[24] Yoshida K, Miki Y (2010). The cell death machinery governed by the p53 tumor suppressor in response to DNA damage. Cancer Sci.

[25] Reisman D, Boggs K (2007). Transcriptional regulation of the p53 tumor suppressor gene: a potential target for cancer therapeutics?. Recent Pat DNA Gene Seq.

[26] Riley T, Sontag E, Chen P (2008). Transcriptional control of human p53-regulated genes. Nat Rev Mol Cell Biol.

[27] Wei Z, Yoshihara E, He N (2018). Vitamin D Switches BAF Complexes to Protect β Cells. Cell.

[28] Mantovani F, Drost J, Voorhoeve PM (2010). Gene regulation and tumor suppression by the bromodomain-containing protein BRD7. Cell Cycle.

[29] Wu M, Li X, Li X (2009). Signaling transduction network mediated by tumor suppressor/susceptibility genes in NPC. Curr Genomics.

[30] Liang Y, Dong B, Shen J (2019). Clinical significance of bromodomain-containing protein 7 and its association with tumor progression in prostate cancer. Oncol Lett.

[31] Park YA, Lee JW, Kim HS (2014). Tumor suppressive effects of bromodomain-containing protein 7 (BRD7) in epithelial ovarian carcinoma. Clin Cancer Res.

[32] Ma J, Niu W, Wang X (2019). Bromodomaincontaining protein 7 sensitizes breast cancer cells to paclitaxel by activating Bcl2antagonist/killer protein. Oncol Rep.

[33] Zhou M, Xu XJ, Zhou HD (2006). BRD2 is one of BRD7-interacting proteins and its over-expression could initiate apoptosis. Mol Cell Biochem.

[34] Park PJ (2009). ChIP-seq: advantages and challenges of a maturing technology. Nat Rev Genet.

[35] Nakato R, Shirahige K (2017). Recent advances in ChIP-seq analysis: from quality management to whole-genome annotation. Brief Bioinform.

[36] Drost J, Mantovani F, Tocco F (2010). BRD7 is a candidate tumour suppressor gene required for p53 function. Nat Cell Biol.

[37] Xu K, Xiong W, Zhou M (2016). Integrating ChIP-sequencing and digital gene expression profiling to identify BRD7 downstream genes and construct their regulating network. Mol Cell Biochem.

[38] Aubrey BJ, Kelly GL, Janic A (2018). How does p53 induce apoptosis and how does this relate to p53-mediated tumour suppression?. Cell Death Differ.

[39] Meng X, Franklin DA, Dong J (2014). MDM2-p53 pathway in hepatocellular carcinoma. Cancer Res.

[40] Lujambio A, Akkari L, Simon J (2013). Non-cell-autonomous tumor suppression by p53. Cell.

[41] Hui Cao, Xiaosong Chen, Zhijun Wang (2020). The role of MDM2-p53 axis dysfunction in the hepatocellular carcinoma transformation. Cell Death Discov.

