# Supplementary material for: BRD7 inhibits tumor progression by positively regulating the p53 pathway in hepatocellular carcinoma
Source: J Cancer. 2021 Jan 1;12(5):1507–19. doi: 10.7150/jca.50293 (PMC7847651; doi:10.7150/jca.50293)
Supplement: Supplementary file 1 — Supplementary figures and table. [file jcav12p1507s1.pdf]

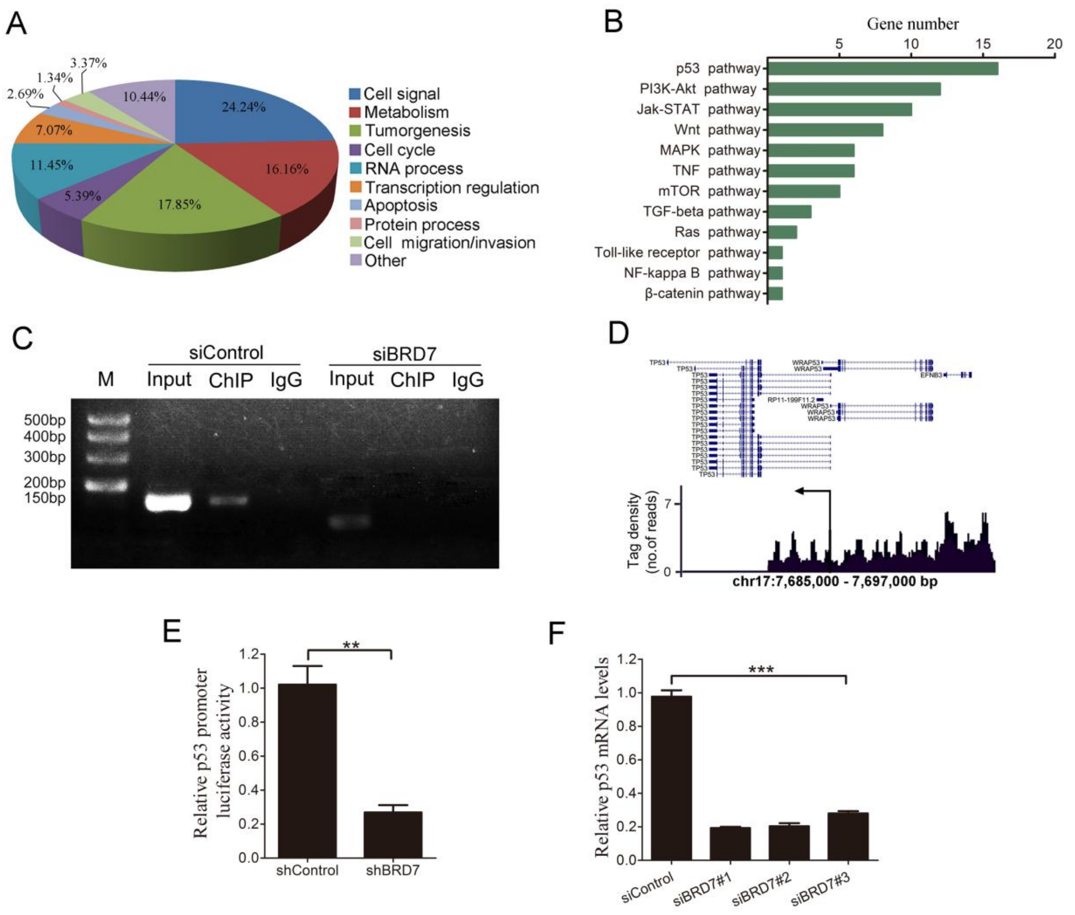

Figure S1. Defining genome-wide BRD7 binding genes by ChIP-sequencing in HepG2 cell lines.

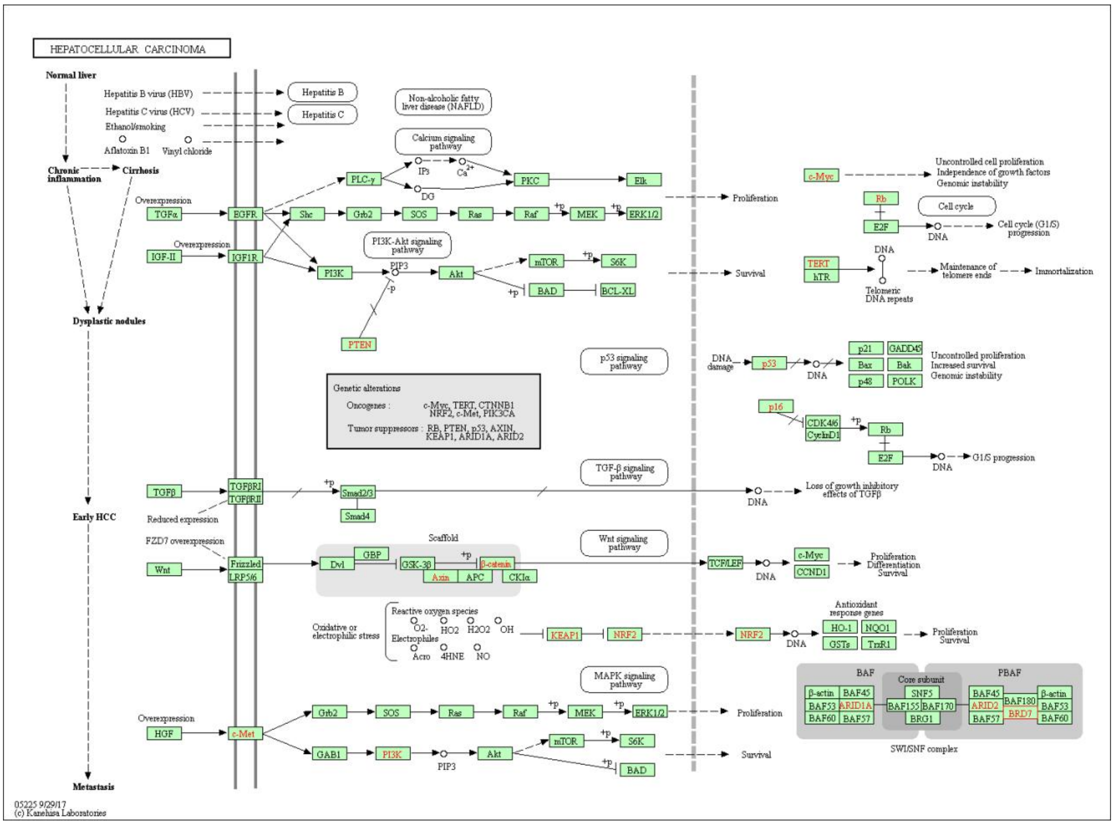

Figure S2. Signaling pathways in HCC, demonstrating that BRD7 is involved in the PI3K-Akt pathway (from TCGA data).

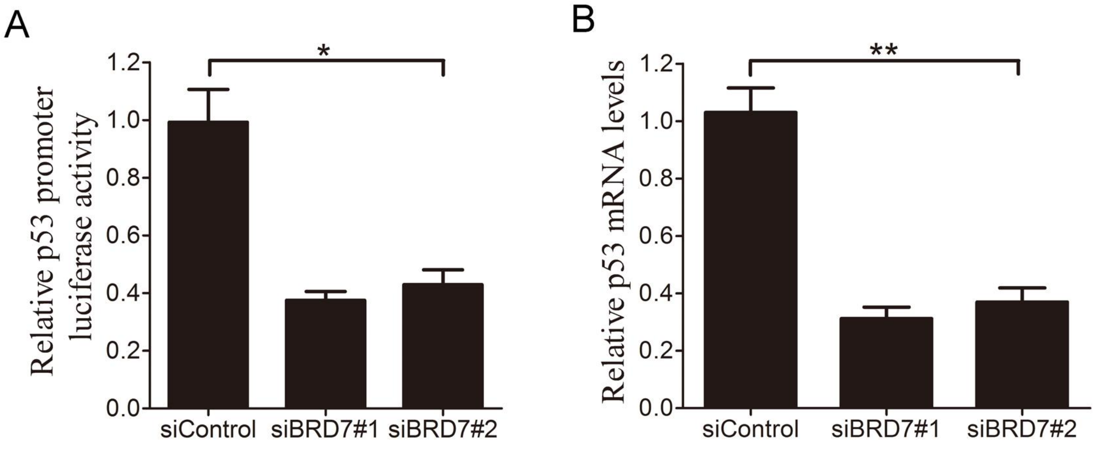

Figure S3. The p53 promoter luciferase activity **(A)** and mRNA expression **(B)** in Bel7402 cells with BRD7 knockdown.

Table S1. Sequences of siRNA

			Sequences(5'-3')
**BRD7**	siRNA-1	sense	GCCUCUCACAAGCUCUUUATT
		anti-sense	UAAAGAGCUUGUGAGAGGCTT
	siRNA-2	sense	GCCAAGAUUAUCCGUAUGUTT
		anti-sense	ACAUACGGAUAAUCUUGGCTT
	siRNA-3	sense	GCCCUACAGUUCUUAUGCATT
		anti-sense	UGCAUAAGAACUGUAGGGCTT
**P53**	siRNA-1	sense	CCCGGACGAUAUUGAACAATT
		anti-sense	UUGUUCAAUAUCGUCCGGGTT
	siRNA-2	sense	CCACCAUCCACUACAACUATT
		anti-sense	UAGUUGUAGUGGAUGGUGGTT
	siRNA-3	sense	GCGCACAGAGGAAGAGAAUTT
		anti-sense	AUUCUCUUCCUCUGUGCGCTT
**Control**	siRNA	sense	UUCUCCGAACGUGUCACGUTT
		anti-sense	ACGUGACACGUUCGGAGAATT
